# Mechanobiology of Epithelia From the Perspective of Extracellular Matrix Heterogeneity

**DOI:** 10.3389/fbioe.2020.596599

**Published:** 2020-11-20

**Authors:** Aleksandra N. Kozyrina, Teodora Piskova, Jacopo Di Russo

**Affiliations:** ^1^Interdisciplinary Centre for Clinical Research, RWTH Aachen University, Aachen, Germany; ^2^Institute of Molecular and Cellular Anatomy, RWTH Aachen University, Aachen, Germany; ^3^DWI – Leibniz-Institute for Interactive Materials, Aachen, Germany

**Keywords:** extracellular matrix, epithelial mechanobiology, basement membrane, interstitial matrix, matrix heterogeneity

## Abstract

Understanding the complexity of the extracellular matrix (ECM) and its variability is a necessary step on the way to engineering functional (bio)materials that serve their respective purposes while relying on cell adhesion. Upon adhesion, cells receive messages which contain both biochemical and mechanical information. The main focus of mechanobiology lies in investigating the role of this mechanical coordination in regulating cellular behavior. In recent years, this focus has been additionally shifted toward cell collectives and the understanding of their behavior as a whole mechanical continuum. Collective cell phenomena very much apply to epithelia which are either simple cell-sheets or more complex three-dimensional structures. Researchers have been mostly using the organization of monolayers to observe their collective behavior in well-defined experimental setups *in vitro*. Nevertheless, recent studies have also reported the impact of ECM remodeling on epithelial morphogenesis *in vivo*. These new concepts, combined with the knowledge of ECM biochemical complexity are of key importance for engineering new interactive materials to support both epithelial remodeling and homeostasis. In this review, we summarize the structure and heterogeneity of the ECM before discussing its impact on the epithelial mechanobiology.

## The Extracellular Matrix of Epithelia

**FIGURE 1:**
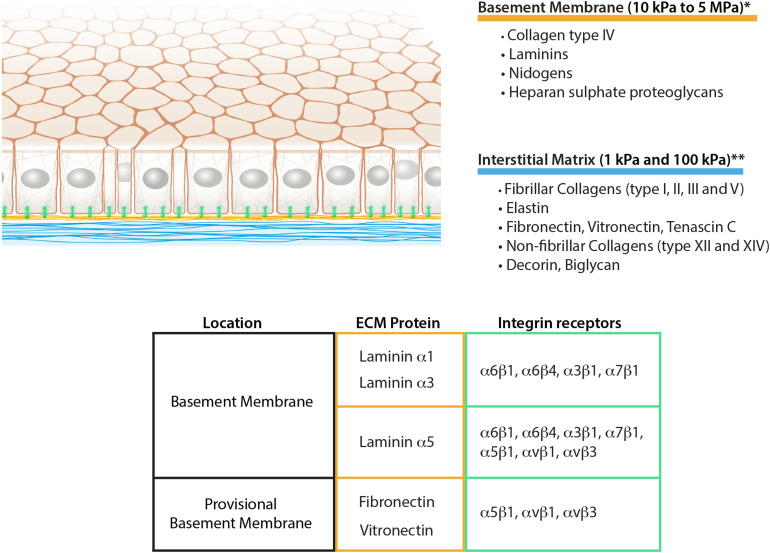
Schematic representation of epithelial ECM, its composition, and mechanical properties. The table summarizes the integrin receptors for the main epithelial laminins together with the fibronectin and vitronectin as aberrant basement membrane components during remodeling. *Grantham et al. (1987), Welling et al. (1995), Candiello et al. (2007), Endlich and Endlich (2012), **Pailler-Mattei et al. (2008), Booth et al. (2012), Peñuela et al. (2018).

**FIGURE 2:**
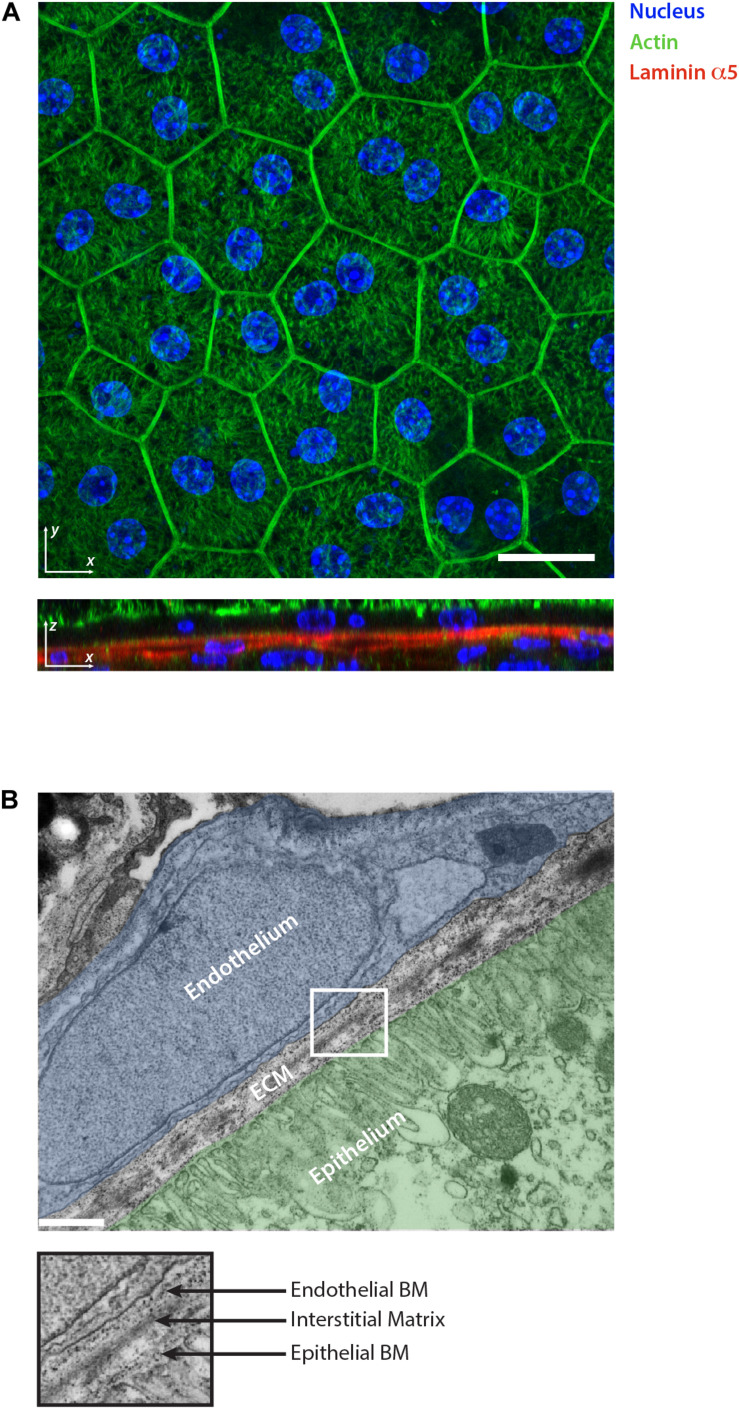
Retinal pigment epithelium as an example of epithelial tissue. **(A)** Whole mount staining of retinal pigment epithelium monolayer for actin, nuclear marker and laminin α5. The 2D visualization of the monolayer surface nicely shows the honeycomb-like structure arrangement of the cells during homeostasis. The optical section reveals the underlining basement membranes of the retinal pigment epithelium and choroidal endothelium stained for laminin α5. Scale bar is 20 μm. **(B)** Electron micrograph showing the ultrastructural organization of the ECM in the retina which separates the epithelium (highlighted in green) from the choroidal endothelium (highlighted in blue). The collagen and elastin rich interstitial matrix is located underneath the basement membrane (BM) of the pigment epithelium. Scale bar is 500 nm.

ECM plays a pivotal role in controlling cell behavior, supporting cell collectives with both biochemical information, and providing a correct mechanical environment (Mouw et al., 2014). Each epithelium is anchored down by the BM which creates a boundary to the cells, separating them from the underlying looser matrix network, called interstitial matrix (IM) or connective tissue (Figures 1, 2). The structural and biochemical distinction of the BM and IM leads to the different levels of contribution in regulating epithelial functions.

The BM is a 60–120 nm thick matrix network composed of collagen type IV, laminins, nidogens, heparan sulfate proteoglycans, and other minor components. Collagen type IV and laminins self-assemble to form two independent networks that interconnect via proteoglycans and nidogens (Hohenester and Yurchenco, 2013). Collagen type IV is a triple-helical molecule that can be assembled from six different chains (α1–α6) forming three distinct isoforms (Bächinger et al., 2010). The most commonly distributed isoform is the [(α1)_2_(α2)], made of two α1 chains and a single α2 chain, but also two other isoforms are present, namely [(α3)(α4)(α5)] and [(α5)_2_(α6)]. The [(α3)(α4)(α5)] subtype is localized in the BM of kidney glomerulus and the alveoli of the lung, whereas [(α5)_2_(α6)] has been found in the epidermis, mammary glands, and epithelium of the alimentary tract (Bächinger et al., 2010).

The laminin network has a much more heterogeneous composition in comparison to collagen type IV. Laminins are cross-shaped heterotrimeric glycoproteins composed of an alpha, a beta and a gamma chain. Five alpha, three beta and three gamma subunits can assemble to form 11 distinct laminin isoforms (Aumailley, 2012). In contrast to collagen type IV, laminin isoform expression varies depending on the tissue. In particular, while the beta and gamma chains are engaged in the formation of the protein network, the C-terminal domain of the alpha chain is kept free for the interaction with the cellular receptors, thus defining the tissue-specific distribution (Aumailley, 2012).

As suggested by the number of different isoforms, the BM represents the most heterogeneous matrix in the epithelia. Tissue-specific localization of laminin isoforms suggests the important role of laminin in controlling specific epithelial functions. Laminin 111 is the most abundant isoform during development and is pivotal in controlling cell polarization and, therefore, tissue shaping (Yurchenco, 2011; Lee and Streuli, 2014). Upon maturation, further isoforms are also expressed instead of laminin 111 in order to maintain the specific epithelium homeostasis (Yurchenco, 2011). One of the most representative examples is laminin 332, well-studied in the epidermal BM. Due to the interaction with specific cellular receptors, it sustains the formation of hemidesmosomes, thus ensuring tissue cohesion upon external mechanical stress (Kiritsi et al., 2013; Rousselle and Beck, 2013). Interestingly, the same protein in cleaved variances or in different splicing versions is also important in sustaining cell-adhesion in the reepithelization of wounds (Wen et al., 2010). Other examples are laminins, containing the α5 chain, such as laminin 511 and 521. The laminin α5 chain is characterized by the peculiarity to carry exposed arginine-glycine-aspartate (RGD) sequences in addition to the cell-binding domains at the C-terminal end (Sasaki and Timpl, 2001). The RGD sequence is known to permit the binding of specific integrin receptors (discussed below) that control cell mechanics, including intercellular adhesion during the endothelial shear-stress response (Di Russo et al., 2017).

As a result of structural distinction, collagen type IV and laminin have different functions in the BM. Collagen monomers are covalently linked to each other, conferring structural stability to the BM and allowing it to withstand tensile strengths (Poschl et al., 2004). In contrast, laminins are considered biologically active components, controlling cell adhesion through the interaction with cellular receptors (Figure 1). Furthermore, it is worth noting that laminins do not self-assemble with covalent bonds, but rather via ionic interactions (Hohenester and Yurchenco, 2013). This supports the idea of the lower force-bearing role of the network and its higher flexibility necessary, for instance, to allow cell crossing during leukocyte migration (Sorokin, 2010). Additionally, it has been shown that the genetic removal of the heparan sulfate proteoglycan perlecan strongly reduces the mechanical impact of BM on epithelial behavior. This suggests that the uncoupling of the cell-interactive laminin network from the mechanical bearing collagen network disrupts the mechanotransduction effect of the matrix (Crest et al., 2017; Chen et al., 2019).

The IM is a distinct ECM layer located underneath the BM. In the homeostatic state, epithelial cells are not in direct contact with the IM, however, the arrangement of its components confers the structure to the epithelium as shown in the skin (Silver et al., 2003). Similar to other connective tissues, epithelial IM is characterized by a fibrillar protein network loosely connected to each other. It is composed of fibrillar collagens (type I, II, III, and V), non-fibrillar collagens (e.g., XII and XIV), glycoproteins including fibronectin, vitronectin and tenascin-C, and proteoglycans such as decorin or biglycan (Mouw et al., 2014; Figure 1). Elastin is also very abundant in the IM and forms an extensive crosslinked network of fibers and sheets. The elastin can be also organized separately from the collagen fibers forming a distinct layer. This is the case in tissues characterized by physiological pressure oscillations that define the mechanical resilience of the matrix. One example of this is found in the Bruch’s membrane of the retinal pigment epithelium where the elasticity of the elastin counteracts the toughness provided by the collagen network (Booij et al., 2010; Figure 2).

Besides the biochemical contribution of the BM to epithelial cell adhesion, some mechanobiological considerations can be deduced only from the ultrastructure of the two matrix layers. Due to the sheet-like organization, the BM is not thought to be flexible, therefore possessing higher mechanical resistance to cellular adhesion. Nevertheless, due to the anatomy of the BM, it is particularly challenging to perform accurate measurement *ex vivo* with the methods currently available. Some attempts of measurements on different BMs have been done using techniques such as atomic force microscopy or micropipette aspiration which provided substantially different results (from 10 kPa to 5 MPa) (Figure 1; Grantham et al., 1987; Welling et al., 1995; Candiello et al., 2007; Endlich and Endlich, 2012). This suggests the high dependency of the measurements on the tissue preparation or the method employed (Grantham et al., 1987; Candiello et al., 2007). In conclusion, new experimental data using less invasive techniques will be necessary to characterize BM properties.

The mechanics of IM on the other hand has been characterized more thoroughly (Guimarães et al., 2020). Its compliance is highly dependent on the relative amount of collagen, elastin, and the level of network crosslinking, which varies according to tissue and age (Birch, 2019). According to several independent measurements, IM Young’s modulus can range between 1 and 100 kPa (Pailler-Mattei et al., 2008; Booth et al., 2012; Peñuela et al., 2018; Figure 1). Additionally, the differing content of polysaccharides such as hyaluronic acid ensures different water retention of the matrix layer, varying its resistance to compressive forces (Lodish et al., 2003).

## Basement Membrane Receptors

To engineer functional biomaterials supporting epithelial tissue, it is crucial to understand the nature of cell-ECM adhesion with its biochemical composition and structures. Epithelial cells are tightly bound to their BM via specific receptors. For the aim of this review, we will mainly focus on integrin receptors due to their major role in regulating cell adhesion and function, while keeping in mind that the importance of non-integrin receptors, including syndecans and the non-muscle dystroglycan complex, should not be underestimated either.

Integrins are a family of transmembrane heterodimeric receptors containing an alpha and a beta chain. As indicated by the name, they integrate the extracellular space with the intracellular cytoskeleton system. In the BM, the alpha chain of laminins defines the specificity for integrin adhesion, commonly with one of the following isoforms: α6β1, α3β1, α6β4 and α7β1 (Pozzi et al., 2017; Figure 1). Integrin α6β1 represents the most promiscuous receptors for laminin isoforms, whereas integrins α3β1 and α6β4 mainly bind to laminin α3 and α5 chains. Finally, α7β1 preferentially interacts with α2 and α5 laminin chains (Belkin and Stepp, 2000). Additionally to the mentioned classical laminin-binding integrins, laminin α5 chain has been shown to promote cell adhesion also via integrins αvβ3, αvβ1 and α5β1 thanks to the RGD sequences present at the N-terminal end (Sasaki and Timpl, 2001; Di Russo et al., 2017; Figure 1).

Integrin receptors contribute to the formation of two main adhesion structures: focal adhesions and hemidesmosomes. Focal adhesions are mechanosensitive multiprotein complexes that connect the integrins to the intracellular actin cytoskeleton. There are no published reports on mature focal adhesion in the homeostatic state of epithelial cells, however, it is known that they appear as soon as remodeling events occur (Underwood et al., 2008; Tarau et al., 2019). This often happens together with the deposition of aberrant ECM proteins such as fibronectin which together with laminins forms a provisional BM (Koivisto et al., 2011; Figure 1). During these processes, epithelial cells lose their polarization and increase adhesion strength to the ECM, thanks to the formation of focal adhesions and actin stress fibers. This allows basal keratinocytes, for instance, to start the process of reepithelization in skin wound healing (Carter et al., 1990; Underwood et al., 2008). Among the laminin-binding integrins, only α3β1 has been suggested to form focal adhesions in epithelia (Carter et al., 1990; Dogic et al., 1998). Furthermore, most epithelial cells also express α5β1 and αvβ3 integrins which play pivotal roles during cell migration (Schiller and Fässler, 2013). In some pathological situations, similar phenotypical changes can also be observed without the presence of a wound but with associated ECM remodeling events. This is the case for the retinal pigment epithelial cells during the progression of age-related macular degeneration (Rickman et al., 2016). Before the disease manifests, aberrant accumulations of ECM can be observed underneath the epithelium. This remodeling suggests the induction of cell phenotypical changes characterized by loss of polarity and the formation of stress fibers (Tarau et al., 2019).

Differently from focal adhesions, hemidesmosomes are epithelial-specific cell-laminin adhesion structures that anchor the cells to the ECM via the keratin intermediate filament cytoskeleton (Walko et al., 2015). Integrin α6β4 heterodimers participate in the formation of hemidesmosomes and together with plakins are connected to the cytokeratin network (Chaudhari and Vaidya, 2014). Hemidesmosomes are crucial for both controlling epithelial tissue homeostasis and mechanical properties including stiffness, stretchability, strength, resilience, and toughness (Nievers et al., 1999; Russell et al., 2004; Ramms et al., 2013; Seltmann et al., 2013). This is demonstrated by the fact that mutations and defects of either hemidesmosome components or one of the binding laminins result in epithelial malfunction and lack of tissue integrity (Kiritsi et al., 2013; Walko et al., 2015). Examples for that are the debilitating disease of the epidermolysis bullosa, characterized by the formation of blisters on the surface of the skin (Kiritsi et al., 2013), or inflammatory lesions caused by hemidesmosomes disruption that can lead to the development of epithelial-derived cancers (Arcangelis et al., 2017).

Aiming to promote specific cell adhesion on engineered biomaterials, scientists have been using ECM-derived synthetic peptides rather than full-length proteins. The first identified sequence which strongly promotes cell adhesion was RGD, initially derived from the fibronectin protein (Mas-Moruno et al., 2016). Currently, different forms of RGD peptides are broadly used to functionalize biomaterials, even though they will only engage a small proportion of integrin isoforms present on epithelial cells (Humphries et al., 2006). Furthermore, the RGD sequence largely supports a remodeling status within the tissue, which might not be the aim of a specific biomaterial. To mimic the ECM while sustaining epithelial homeostasis, BM-derived peptides would be more advantageous. This can be achieved by testing and optimizing a combination of some of the many available laminin-derived peptides (Kikkawa et al., 2013) and, ultimately, by developing a hemidesmosome-supporting material to provide integrity and mechanical resilience to the epithelia.

## Epithelial Mechanics

### From Single Cell to Collective

Additionally to the cell-ECM adhesion heterogeneity, the impact of cellular mechanotransduction and the length scale to which forces are sensed has to be taken into consideration. Due to their position between body compartments, epithelia need to withstand external forces and respond accordingly (Califano and Reinhart-King, 2009; Tschumperlin et al., 2009; Heisenberg and Bellaïche, 2013). This is achieved through their high number of intercellular adhesion structures mainly organized in adherents and tight junctions (Bazellières et al., 2015). In the last few years, it became clear that in addition to ensuring tissue barrier, these intercellular interactions make epithelia a biochemical and mechanical continuum (Trepat and Sahai, 2018; Shellard and Mayor, 2019). In particular, mechanical properties and behavior are difficult to explain, looking at a single cell forming the tissue and without considering epithelia as a functional cell collective.

From the biomechanical point of view, several models have been proposed to explain single-cell mechanical behavior (Rajagopal et al., 2017). Cells can be depicted as systems with elastic borders (membrane) and homogeneous internal components made of viscous, viscoelastic, or elastic matter (cytoplasm) (Karcher et al., 2003). However, these models are not useful in explaining the mechanical properties of cells because they neglect the internal microstructures and the interaction with the cellular environment. One of the most fitting ideas describing the dynamic mechanical equilibrium of cells is the tensegrity model, introduced by Donald Ingber (Ingber, 2003). The model considers cellular architecture under tensional integrity or “tensegrity” which defines cell shape in each cell condition. Tensional forces created by cytoskeletal microfilaments (actin) are balanced by compression borne by interconnected structural filaments, including internal microtubule struts and intermediate filaments that are connected to cell-ECM adhesion structures (Ingber et al., 2014). The cell shape represents the total equilibrium of inner and outer forces as an effective work of the cytoskeleton network. Therefore, this model offers a good explanation as to why cells have a round shape when floating, or exhibit greater spread on hard substrata compared to soft.

This dynamic equilibrium of forces is not only important for a single cell during the division and migration but also plays a crucial role at the multicellular level (Vishwakarma and Di Russo, 2019). In epithelia, individual cells balance cell-ECM traction forces with the adhesion to neighboring cells, therefore creating cell-cell stresses spread throughout the whole tissue. In this respect, the above-described model might be extended to a “collective tensegrity.” Thus, from the physiological point of view, this is of high relevance to understanding the mechanical stimuli affecting the epithelia at a multicellular level or mesoscale (Trepat and Sahai, 2018). Intriguingly, if the ECM is carefully removed without interfering with cell-cell interaction, epithelia are not rounding up as might be expected but instead changing the specialized tissue morphology and function. This supports the importance of ECM in controlling epithelial mechanobiology (Banerjee et al., 1977). Moreover, a recent study on drosophila leg development has demonstrated that ECM plays a key role in regulating epithelial tensegrity. It was shown that the peripodial epithelium which defines the imaginal leg disc, builds up a multicellular tension through the mechanical constrain of the BM. Nevertheless, later in development, the monolayer has to detach from its BM to overcome the matrix constrain and lose its tensegrity, thus allowing the epithelial rupture and retraction crucial for leg elongation (Proag et al., 2019). In conclusion, the mechanical properties of epithelia as a complex system cannot be considered without the connection to the specific ECM environment.

To dissect the relationship between cell-ECM traction forces and cell-cell stress, experiments using a minimal model of epithelial tissue were conducted (Maruthamuthu et al., 2011). The analyses of pairs of interacting epithelial cells revealed not just a force misbalance from the single-cell point of view, but rather equilibrium within the whole system. As a consequence of the formation of adherens junctions, epithelial cells interconnect to each other with an endogenous force of about 100 nN (Maruthamuthu et al., 2011). With the increasing number of cells in the system, clusters and sheets of cells show the same behavior. Therefore, with increasing cell numbers the traction forces raise only at the colony peripheries and the stresses redistribute or dissipate within the whole cell collective (Maruthamuthu et al., 2011; Sunyer et al., 2016; Shellard and Mayor, 2019). Altering cell-ECM adhesion also impacts the intercellular stresses as a result of the new force balance with the substrate, supporting the tensegrity model. To be noted, not only the mechanical properties (e.g., stiffness) of the ECM but also its biochemical composition alone affects force redistribution in the collective, indicating the significance of ECM complexity in epithelial mechanics (Maruthamuthu et al., 2011; Vishwakarma and Di Russo, 2019). With regards to this ability of epithelia to balance forces, an interesting phenomenon has been observed in keratinocytes: in the process of reepithelization in wound healing, basal keratinocytes might encounter a region of low adhesiveness while migrating on a heterogeneous matrix. It has been described that in order to maintain tissue integrity, keratinocytes monolayers can bridge non-adhesive ECM regions, increasing intercellular stresses through cooperative traction forces at the monolayer edges (Vedula et al., 2014).

### ECM, Epithelial Dynamics, and Morphogenesis

Epithelia are highly dynamic tissues with active cell division, cell mingling, and replacement of damaged or dead cells (Vishwakarma and Di Russo, 2019). These natural rearrangements lead to a redistribution of forces and, thus, a mechanical heterogeneity of the whole epithelia (Vishwakarma and Di Russo, 2019). This is also a consequence of the organization in coordinated cell-packs within the tissue, thus creating only a local order within otherwise heterogeneous tissue (Garrahan, 2011). Experimental evidence of epithelial monolayer traction force microscopy and its 3D plotting have shown that monolayer stress distribution is a rugged landscape where peaks (high-stress regions) or valleys (low-stress regions) are equivalent to the region of cellular coordination where forces are redistributed within cell-packs (Tambe et al., 2011). These packs can be characterized by correlating the force vectors or velocities vectors, in the presence of cell movements. In specific environmental conditions, these packs possess a critical size below which cells can coordinate stress. The size of coordination largely depends on ECM mechanical features as similarly shown by Maruthamuthu V. and colleagues for smaller cell clusters (Maruthamuthu et al., 2011; Vishwakarma et al., 2018). The universality of this phenomenon was shown with MDCK cells (tubular kidney epithelium) and HaCaT cells (keratinocytes) which are phenotypically and functionally different but both are able to cooperate on a scale of 10–15 cell diameters (Tambe et al., 2011; Vishwakarma et al., 2018). This stress heterogeneity of the monolayer has been shown to exist both *in vitro* and *in vivo* (Garrahan, 2011; Mongera et al., 2018) and a growing amount of data supports its important role in controlling epithelial functions (Vishwakarma and Di Russo, 2019; Vishwakarma et al., 2020). For example, it has been proposed that the ability of epithelial cells to coordinate in packs may play a role in regulating the extrusion of apoptotic cells from monolayers (Saw et al., 2017).

Recent data *in vivo* also strongly support the idea of the connection between local ECM heterogeneity and this epithelial mechanical anisotropy (Crest et al., 2017; Sui et al., 2018; Fiore et al., 2020). It has been shown that during carcinoma development, mutated cells more efficiently cooperate with each other, thus creating a local increase of tissue stiffness. Here, the mechanical contribution of the epidermal BM in the tumor region plays a more important role compared to the actual epithelial stiffness in regulating tumor morphology (Fiore et al., 2020). Additionally, *in vitro* experiments using 3D epithelial spheroids and artificial matrices have shown the connection between integrin receptors clustering and ECM stiffness (Chaudhuri et al., 2014). The alteration of the epithelial phenotype is supported only when the ECM stiffness increases without an increase of ligand density. In particular, the failure of integrin α6β4 clustering and hemidesmosomes formation induces epithelial malignancy pathways. Altogether, these data highlight the underestimated complex relation between the ECM mechanics and specific biochemical heterogeneity in controlling epithelial phenotype.

Growing body of observation from developmental processes involving epithelial morphogenesis has shown that ECM patterning plays a key role in controlling local tissue mechanics (Crest et al., 2017; Sui et al., 2018). Similar to what has been shown during carcinoma expansion (Fiore et al., 2020), in drosophila egg chamber elongation and wing imaginal disc formation, the epithelial folding is mainly regulated by ECM resilience. Surprisingly, polarized cellular actomyosin contraction plays a minor role in epithelial morphogenesis, but rather the local reduction of BM density drives the monolayer mechanical anisotropy (Sui et al., 2018).

During dynamic cell rearrangements such as in development (Mongera et al., 2018), cells possess a certain persistence of motion related to migration and preferred cell shape as a result of the balance between the specific adhesion and tension forces (Bi et al., 2015). A shift of these parameters can define a monolayer as solid-like or fluid-like and leads to transitions that have been compared to glass transition occurring in a supercooled fluid or dense particulate matter (Garrahan, 2011). The solid-like state of the epithelia is characterized by nearly homogeneous shapes and constant position of cells with little fluctuations, as in homeostatic tissue. However, certain conditions might lead to unjamming and reshuffling of the monolayer (Malinverno et al., 2017), leading to shape and coordination heterogeneity of the whole tissue. Shape heterogeneity appears as a result of dynamic monolayer reconfiguration and produces high inner energy that provides the epithelium with specific mechanical properties. This could predetermine the phase transition between the jammed and unjammed states of cell sheets (Park et al., 2016). ECM remodeling has been shown to regulate the conversion of cell shape in epithelia during development (Sui et al., 2018). In the elongation of drosophila wings and legs, epithelial cells undergo columnar to cuboidal shape transformation to allow enlargement of cells area and thus a collective expansion of the tissue. This process is driven by the fine regulation of specific proteinase expression which locally changes the ECM leading to alteration of actomyosin contraction in the cells (Sui et al., 2018). Matrix mechanical gradient commands not only the shape of the cells but also its eccentricity and the orientation of division, suggesting a link between ECM anisotropy and epithelial local unjamming crucial for tissue morphogenesis (Mongera et al., 2018; Chen et al., 2019).

**FIGURE 3:**
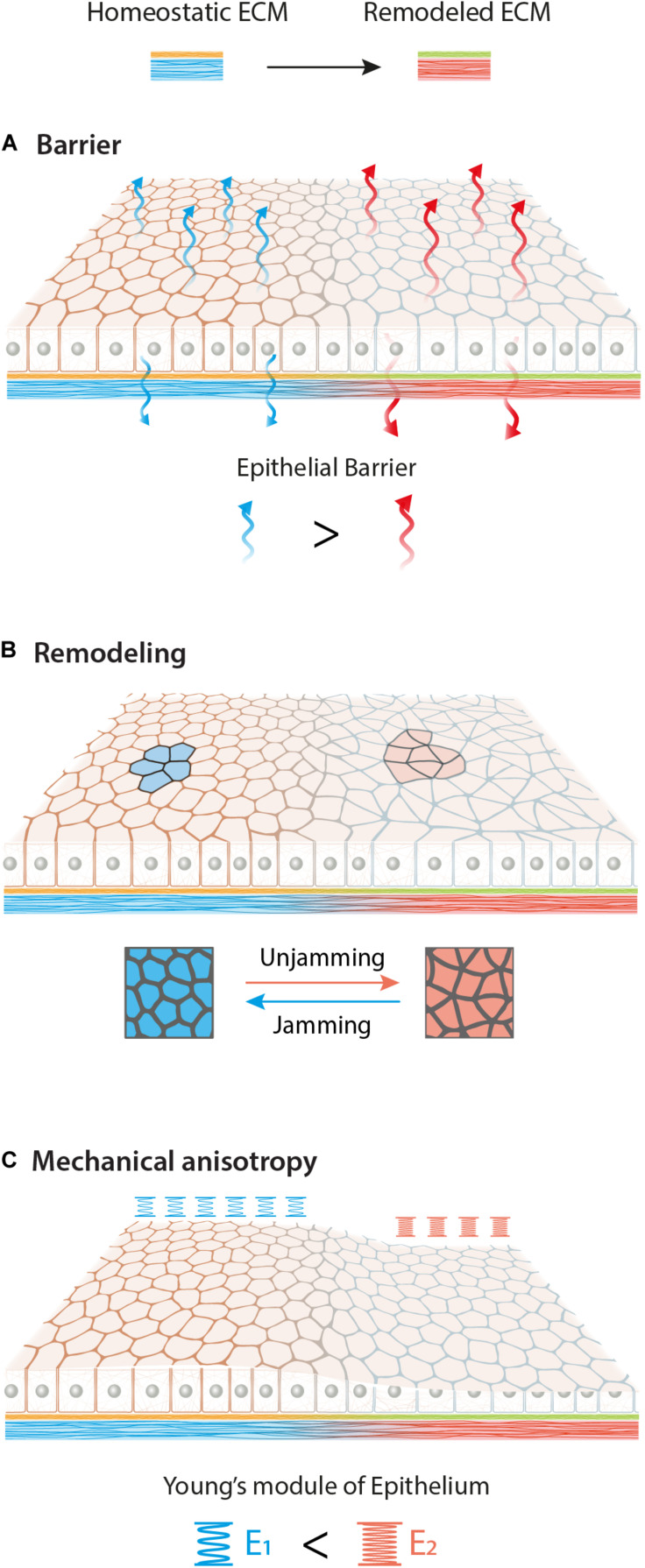
Graphical overview of possible effects of ECM biochemical (yellow to green) and mechanical (blue to red) remodeling on epithelial tissue. Different adhesion conditions may affect epithelia in various aspects such as barrier function **(A)**, remodeling **(B)** and mechanical anisotropy **(C)**. Here we schematize an increase of permeability, a jamming-unjamming transition and a stiffening of the monolayer upon new ECM.

In the homeostatic state of the adult epithelia, within overcrowded regions, external forces tend to redistribute in order to be balanced for each particular cell. As a result, most of the cells possess a hexagonal shape and arrange themselves in honeycomb-like structures as a representation of an energetically beneficial state (Figure 2A; Bi et al., 2015). Using computer simulations of the vortex model, it has been shown that the transition between jammed and unjammed status can be modeled and predicted using an adimensional shape factor obtained combining cell area and perimeter ($q=P/\sqrt{A}$) (Bi et al., 2015). The different predictions were used to obtain a jamming phase diagram, suggesting the presence of a threshold of intercellular stresses and traction forces up to which epithelia stay in a jammed status (Park et al., 2016). As discussed above, the emerging role of cell jamming-unjamming transition was highlighted in development but also appears in the context of asthma and cancer (Park et al., 2015; Mongera et al., 2018; Palamidessi et al., 2019). It has been shown that the epithelial monolayer develops a different level of traction forces and stresses in asthmatic vs. non-asthmatic patients. Cells from asthmatic patients showed a lower threshold to undergo phase transition, making the epithelium hypersensitive to stresses (Park et al., 2015). Differences in traction forces can also increase due to ECM mechanical and biochemical remodeling. Therefore, it is reasonable to think that local ECM heterogeneity has a central role in controlling the threshold of phase transition also in diseases (Tambe et al., 2011; Ladoux and Mege, 2017; Vishwakarma and Di Russo, 2019; Figure 3). Tissue morphogenesis and cancer development are also characterized by the collective migration of epithelial cells (Rørth, 2012). This inherent ability of all epithelia is possible due to the intercellular transmission of forces which allows for the efficient coordination of their movement during migration. Upon cell movement, the increased tension at tight junctions with the neighbor cell leads to the formation of an intracellular Rac1 gradient (Das et al., 2015), which makes each individual cell align and migrate toward the net force, thus minimizing intercellular shear stresses in a process called plithotaxis (Trepat and Fredberg, 2011). As a consequence of plithotaxis, epithelia are able to respond to environmental changes on a larger scale and more efficient scale compared to single cells. This has been nicely demonstrated in the ability of monolayers to move toward stiffness gradients of ECM substrata (collective durotaxis) (Sunyer et al., 2016) underlining the functional importance of supracellular force transmission for epithelial function (Shellard and Mayor, 2019). During the onset of collective monolayer migration, the length scale of force propagation is directly correlated to the amount of stress present in the epithelia. It has been shown that stiffer substrata and thus larger distances of force correlation, induce a lower number of leader cells to guide monolayer migration (Vishwakarma et al., 2018). Long-range force transmission also has the role of dissipating mechanical stress as shown by monolayer deformations that emerge right after wound closure and propagate across the whole epithelium as decaying waves (Rodríguez-Franco et al., 2017). In conclusion, also in collective migration, the complex relation of ECM mechanical and biochemical signals has a direct effect in defining the intercellular stress distribution and its length scale.

## Final Remarks

Understanding ECM composition in different biological processes and its implication for epithelial mechanobiology is fundamental to suitably engineer biomaterials to support epithelial tissues. For example, distinct laminin isoforms in the BM or the presence of aberrant matrix proteins such as fibronectin, dramatically differ in controlling cell adhesion, traction forces, and thus functions. The same is valid for variable stiffnesses provided by the IMs. Nevertheless, it is still unclear how the nature of ECM ligands and their density in relation to the stiffness may affect mechanotransduction processes. Till now mechanobiological studies have been conducted in developmental contexts or using *in vitro* systems. Many open questions still need answers for the mechanobiological impact resulting in the dramatic changes occurring during epithelial aging and disease.

Altogether different ECM biochemical and mechanical cues strongly influence epithelial functions. New ECM composition may lead to an alteration of the epithelial barrier, monolayer remodeling and the formation of local mechanical anisotropy (Figure 3). Finally, it is important to look at epithelial behavior at the multicellular mesoscale level, due to their collective behavior and their ability to respond and “sense” the environment at mm rather than μm scale.

## Author Contributions

ANK and JDR wrote and corrected the manuscript. ANK, TP, and JDR prepared the illustrations and proofread the manuscript. All authors contributed to the article and approved the submitted version.

## Conflict of Interest

The authors declare that the research was conducted in the absence of any commercial or financial relationships that could be construed as a potential conflict of interest.
